# Successful Use of Extracorporeal LVAD for Cardiogenic Shock with Aortic Valve Regurgitation as Bridge to Surgery

**DOI:** 10.70352/scrj.cr.25-0343

**Published:** 2025-07-26

**Authors:** Takura Taguchi, Takuji Kawamura, Daisuke Yoshioka, Shunsuke Saito, Ai Kawamura, Yusuke Misumi, Shigeru Miyagawa

**Affiliations:** Department of Cardiovascular Surgery, University of Osaka Graduate School of Medicine, Suita, Osaka, Japan

**Keywords:** extracorporeal left ventricular assist device, cardiogenic shock, aortic valve regurgitation, case report

## Abstract

**INTRODUCTION:**

Intra-aortic balloon pumping (IABP), Impella, and veno-arterial extracorporeal membrane oxygenation (VA-ECMO) are common percutaneous devices used to manage hemodynamic instability in patients with cardiogenic shock. These devices play a critical role in providing circulatory support. However, they may fail to achieve sufficient left ventricular unloading in patients with aortic valve regurgitation (AR), potentially complicating treatment strategies. In such challenging cases, an extracorporeal left ventricular assist device (LVAD) may serve as an effective alternative solution.

**CASE PRESENTATION:**

A 61-year-old man presented with heart failure and cardiogenic shock, further complicated by AR. Despite intensive inotropic therapy, his condition deteriorated, leading to significant hepatic and renal dysfunction. Echocardiography revealed left ventricular dysfunction with an ejection fraction of 23.5%, as well as moderate aortic, mitral, and tricuspid valve regurgitation. Initial management with VA-ECMO proved inadequate, necessitating the implantation of an extracorporeal LVAD. This intervention resulted in marked improvements in hemodynamics and multi-organ function. Subsequently, the patient underwent successful surgical procedures, including aortic valve replacement, mitral and tricuspid annuloplasty, and pulmonary vein isolation. He was discharged on day 51.

**CONCLUSIONS:**

This case highlights the challenges in managing cardiogenic shock with AR, where conventional devices like IABP, Impella, and VA-ECMO may exacerbate the condition. The use of an extracorporeal LVAD provided effective left ventricular unloading, enabling successful preoperative optimization and surgery. This case supports the utility of LVAD as a bridge to surgery in patients with cardiogenic shock and AR, suggesting a need for further research into optimal management strategies in such complex cases.

## Abbreviations

AR
: aortic valve regurgitation
AVR
: aortic valve replacement
CI
: cardiac index
EF
: ejection fraction
IABP
: intra-aortic balloon pumping
LVAD
: left ventricular assist device
LVEDD/SD
: left ventricular end-diastolic dimension/end-systolic dimension
PAP
: pulmonary artery pressure
PAWP
: pulmonary artery wedge pressure
RAP
: right atrial pressure
STS
: society of thoracic surgeons
SvO_2_
: mixed venous oxygen saturation
VA-ECMO
: veno-arterial extracorporeal membrane oxygenation

## INTRODUCTION

IABP, Impella, and VA-ECMO are useful percutaneous devices for improving hemodynamics in patients with cardiogenic shock.^1)^ However, sufficient left ventricular unloading cannot be achieved in AR cases, making it challenging to provide adequate support.^2)^

We report a case of cardiogenic shock with AR, where the insertion of a temporary extracorporeal LVAD successfully improved hemodynamic stability, facilitated left ventricular unloading, and allowed for effective preoperative optimization.

## CASE PRESENTATION

**Fig. 1:**
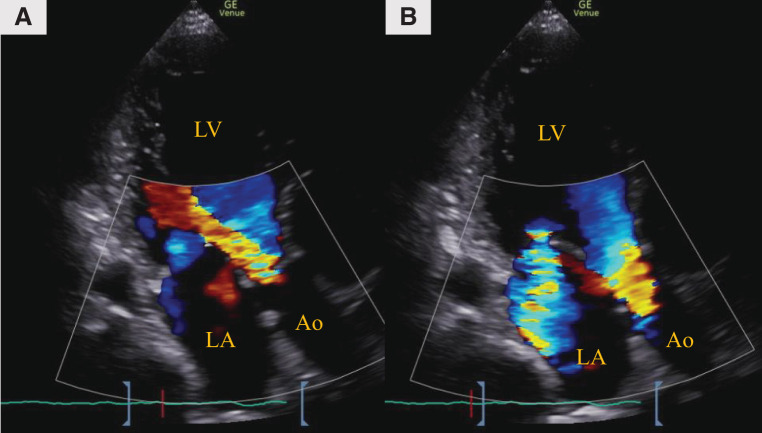
Preoperative transthoracic echocardiography. (**A**) Aortic valve regurgitation and (**B**) Mitral valve regurgitation. Ao, aorta; LA, left atrium; LV, left ventricle

A 61-year-old man with a body surface area of 2.00 m^2^ was receiving treatment for heart failure, presenting with exertional dyspnea. Despite inotrope administration, his hemodynamics did not improve, and hepatic and renal dysfunction progressed, leading to his transfer to our hospital. With dobutamine infusion at 5 γ, the patient’s heart rate was 120 bpm, blood pressure was 116/70 mmHg, and atrial flutter was observed on electrocardiogram. Laboratory test results showed the following: prothrombin time-international normalized ratio 8.6, creatinine 1.91 mg/dL, aspartate aminotransferase 838 U/L, alanine aminotransferase 963 U/L, total bilirubin 2.1 mg/dL, and brain natriuretic peptide 1312.4 pg/mL. Echocardiography showed an LVEDD diameter and LVESD diameter of 69.9 and 62.2 mm, respectively, with an EF of 23.5%. Moderate aortic, mitral, and tricuspid valve regurgitation was also observed (**Fig. 1**). Right heart catheterization revealed PAP: 57/35 (44) mmHg; RAP: 19 mmHg; PAWP: 35 mmHg; CI: 0.9 L/min/m^2^; and SvO_2_: 35.2%. The STS score-calculated operative mortality for isolated AVR was 18.1%.

**Fig. 2:**
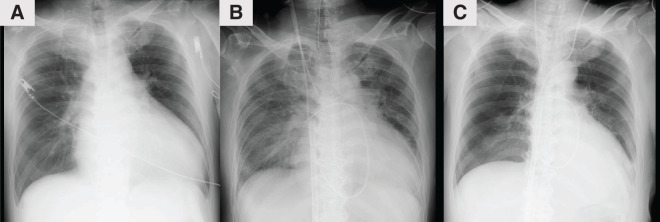
Change over time in chest X-ray. Serial chest X-rays showing changes over time: (**A**) at admission, (**B**) prior to LVAD implantation, and (**C**) 5 days after LVAD implantation. LVAD, left ventricular assist device

**Fig. 3:**
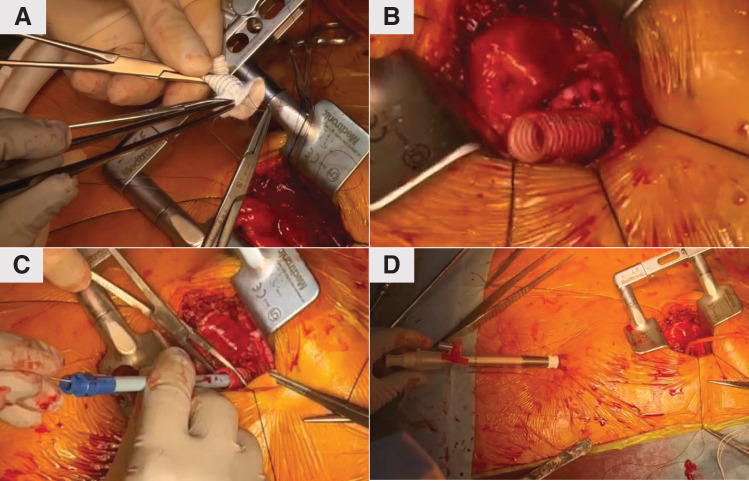
Extracorporeal left ventricular assist device surgery technique. (**A**) A cuff was made from an artificial blood vessel (J-graft, 9 mm) and felt. A thread was sutured to the apex and passed through the cuff. (**B**) The cuff was sutured at the apex. (**C**) A guidewire was inserted through the cuff into the left ventricle, followed by the insertion of a dilator. (**D**) The guidewire was advanced toward the left costal area, and a 19-Fr cannula (Biomedicus; Medtronic, Minneapolis, MN, USA) was inserted into the left ventricle.

**Fig. 4:**
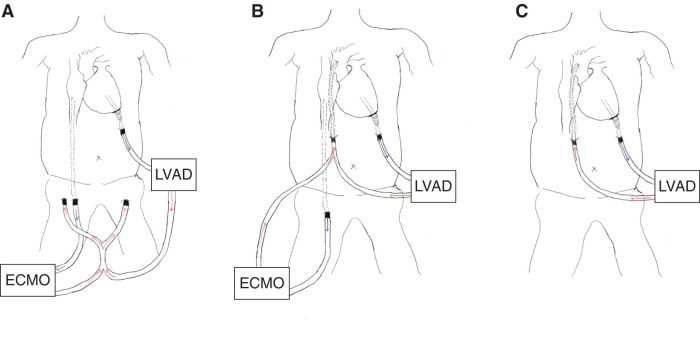
Schematic illustration of extracorporeal LVAD setup and flow configuration. (**A**) Preoperative setup before surgical valve procedures, showing combined support with extracorporeal LVAD and VA-ECMO. Blood is drained from the left ventricular apex and returned via bilateral femoral arteries. (**B**) Configuration immediately after aortic valve replacement, mitral annuloplasty, and tricuspid annuloplasty, illustrating conversion of the outflow cannulation from the femoral arteries to the ascending aorta. (**C**) Configuration after weaning from VA-ECMO, showing continued support with the LVAD alone. LVAD, left ventricular assist device; VA-ECMO, veno-arterial extracorporeal membrane oxygenation

**Fig. 5:**
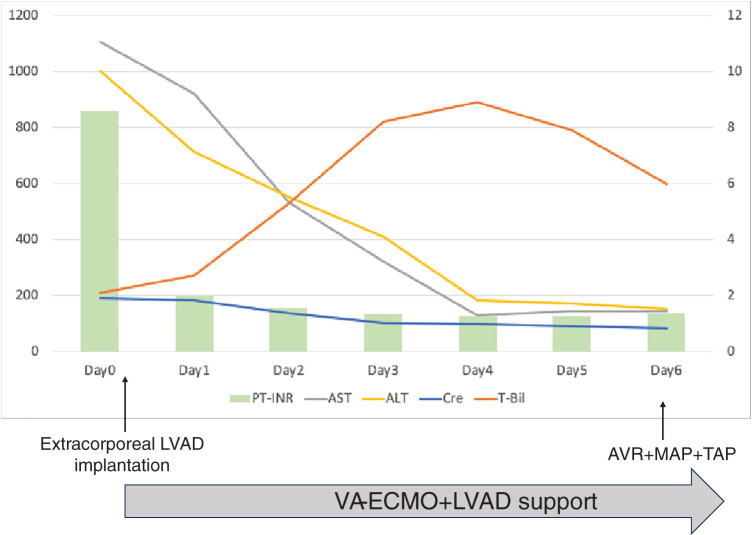
Time-course changes in key biomarkers following initiation of VA-ECMO and extracorporeal LVAD support. The left Y-axis indicates AST (U/L) and ALT (U/L). The right Y-axis indicates PT-INR, serum creatinine (mg/dL), and T-Bil (mg/dL). ALT, alanine aminotransferase; AST, aspartate aminotransferase; AVR, aortic valve replacement; Cre, creatinine; LVAD, left ventricular assist device; MAP, mitral annuloplasty; PT-INR, prothrombin time-international normalized ratio; TAP, tricuspid annuloplasty; T-Bil, total bilirubin; VA-ECMO, veno-arterial extracorporeal membrane oxygenation

VA-ECMO was inserted via the femoral artery with a 14-Fr cannula (PCKC; Senko Medical, Tokyo, Japan) and the femoral vein with a 24-Fr cannula (PCKC), and the patient was admitted to the ICU. However, a chest X-ray taken 6 h later revealed worsening pulmonary congestion (**Fig. 2A** and **2B**), prompting the decision to implant an extracorporeal LVAD. Under VA-ECMO support, a left thoracotomy was performed in the seventh intercostal space to implant the extracorporeal LVAD. Using a 9-mm J-graft (Japan Lifeline, Tokyo, Japan), a skirt-like cuff was created by suturing a strip of felt circumferentially to one end of the graft. At the center of the left ventricular apex, a dimple was identified, and 8 evenly spaced mattress sutures with 3-0 SH-1 Prolene (Ethicon, Raritan, NJ, USA) were placed around it. Each suture was passed through the felt portion of the cuff to secure the conduit firmly to the apex. After completing the anastomosis, a 19-Fr cannula (Biomedicus; Medtronic, Minneapolis, MN, USA) was inserted through the conduit into the left ventricular cavity and connected to the extracorporeal circuit (**Fig. 3**). The outflow of the extracorporeal LVAD was connected to the outflow of the VA-ECMO. An additional 19-Fr cannula (Biomedicus) was inserted into the contralateral femoral artery opposite to the VA-ECMO arterial cannula. From this merged outflow, blood was distributed via a bifurcated Y-connector to both femoral arteries (**Fig. 4A**). Hemodynamic parameters improved with VA-ECMO flow at 4.0 L/min and LVAD flow at 4.2 L/min, resulting in PAP: 24/14 (17) mmHg, RAP: 8 mmHg, PAWP: 10 mmHg, CI: 2.74 L/min/m^2^, and SvO_2_: 74.4%. Hepatic and renal dysfunction, coagulopathy, and pulmonary congestion improved (**Figs. 2C** and **5**). The STS score-calculated operative mortality for isolated AVR improved to 9.5%.

On the fifth day after LVAD implantation, AVR (Inspiris Resilia 23 mm; Edwards Lifesciences, Irvine, CA, USA), mitral annuloplasty (Physio II 32 mm; Edwards Lifesciences), tricuspid annuloplasty (Physio tricuspid 30 mm; Edwards Lifesciences), pulmonary vein isolation, and left atrial appendage closure were performed. Due to the difficulty in weaning from cardiopulmonary bypass, a 14-mm Hemashield graft (Getinge, Gothenburg, Sweden) was anastomosed to the ascending aorta, and a 21-Fr cannula (Biomedicus) was inserted into this graft to provide a common outflow route for the VA-ECMO and LVAD (**Fig. 4B**). The patient was returned to the ICU with the VA-ECMO and LVAD system. VA-ECMO was weaned on POD 3 (**Fig. 4C**), and the LVAD was removed on day 14. The outflow cannula inserted into the ascending aortic graft had been tunneled through the skin; it was removed, and the graft was closed by direct suture ligation. For the left ventricular apical conduit, a repeat left intercostal thoracotomy was performed to expose the graft, the cannula was removed, and the conduit was closed by suture ligation. Postoperative echocardiography showed LVEDD/SD of 51/41 mm and EF of 44%. The patient was discharged on POD 51.

## DISCUSSION

Cardiogenic shock with AR often presents challenges in providing adequate circulatory support using conventional devices. In particular, devices such as IABP, Impella, and VA-ECMO may cause retrograde flow from the aorta into the left ventricle, leading to insufficient unloading.^1,2)^ Therefore, the optimal treatment strategy for patients with cardiogenic shock and AR remains controversial, and individualized treatment approaches are required.

In this case, percutaneous devices such as IABP and Impella were considered inadequate due to the potential for worsening AR and insufficient left ventricular unloading. Therefore, an extracorporeal LVAD was chosen. While continuous LV drainage may theoretically increase the regurgitant volume in aortic regurgitation, extracorporeal LVAD drainage from the apex does not require transvalvular placement and may have a relatively lower risk of worsening regurgitation compared to devices such as Impella, which traverse the aortic valve and can impair leaflet coaptation. In this case, the net unloading effect achieved with the LVAD outweighed any increase in regurgitant volume, resulting in improved hemodynamics and resolution of pulmonary congestion. The overall unloading effect provided by the LVAD was sufficient to improve hemodynamics and resolve pulmonary congestion, despite any potential increase in regurgitant volume.

While AVR is necessary for patients with AR and cardiogenic shock, proceeding with surgery without preoperative optimization may increase the risk of intraoperative and postoperative complications. Although there are reports of improved outcomes with transcatheter AVR for AR, it is not a standard method.^3,4)^ Despite the progression of hepatic and renal dysfunction and coagulopathy, the hemodynamic stabilization achieved by LVAD support allowed for preoperative optimization. LVAD support stabilized the patient’s hemodynamics, enabling safe surgery and early weaning from VA-ECMO and LVAD postoperatively. This suggests that even in patients with severe organ dysfunction, a staged approach using LVAD may be effective in reducing the risks associated with surgery.

There is a report that LV unloading is effective with left atrial VA-ECMO in shock^5)^ with AR.^6)^ In this case, surgical intervention on the mitral and tricuspid valves and the absence of a groin cannula made extracorporeal LVAD a suitable choice, facilitating postoperative rehabilitation. Future research should develop guidelines for managing cardiogenic shock with AR, including optimal timing and device selection. The combined use of VA-ECMO and LVAD represents a promising strategy, offering tailored solutions for severe heart failure with AR or left ventricular dysfunction, potentially improving patient outcomes.

## CONCLUSIONS

We have reported a case of cardiogenic shock with aortic regurgitation where conventional support devices were insufficient. Extracorporeal LVAD provided effective left ventricular unloading, enabling preoperative optimization and successful surgery. This case supports its use as a bridge-to-surgery strategy in such complex scenarios.

## ACKNOWLEDGMENTS

We thank the medical, nursing, and clinical engineering staff of Osaka University Hospital for their valuable support in the management of this patient.

## DECLARATIONS

### Funding

The authors received no specific funding for this work.

### Authors’ contributions

TT drafted the manuscript and collected the clinical data.

TK supervised the manuscript preparation and revised it critically for important intellectual content.

DY, SS, YM, and SM participated in the patient’s surgical treatment and postoperative management.

All authors read and approved the final manuscript.

### Availability of data and materials

The data underlying this article will be shared upon reasonable request to the corresponding author.

### Ethics approval and consent to participate

This study was approved by the Institutional Review Board of Osaka University Hospital (approval no. 16105).

### Consent for publication

The authors confirm that written informed consent for submission and publication of this case report, including images and associated text, has been obtained from the patient in line with COPE guidance.

### Competing interests

The authors declare that they have no competing interests.
